# Comment on Pituitary Tumour Apoplexy as Cause of Death of Simonetta Vespucci

**DOI:** 10.1002/edm2.70334

**Published:** 2026-09-02

**Authors:** Alexis Demas

**Affiliations:** ^1^ ARGOS (Arts, Research and Gestures Observed Scientifically), Neurosciences and Arts Paris France

Dear Editor,

Nardelli and colleagues propose pituitary tumour apoplexy as the cause of Simonetta Vespucci's death [1]. Their reconstruction is careful and the differential they set out is appropriately open. If the diagnosis is granted, the historical record may permit a more specific anatomo‐clinical reconstruction than the one offered, and may resolve an apparent inconsistency within it.

The authors argue that the adenoma expanded through the sphenoid sinus and nasal cavities while sparing the visual pathways, noting the absence of any recorded visual disturbance. Yet they also invoke a series of adolescent patients in which, on their reading, visual impairment including blurred vision and photophobia affected two‐thirds [2], and conclude that this likely afflicted Simonetta because she rested in darkness. The two claims are difficult to hold together.

The difficulty is one of classification. Photophobia is not a chiasmatic sign. Compression of the optic pathways produces field loss or reduced acuity; photophobia arises from meningeal irritation and trigeminovascular activation. These localise to different territories, and grouping them as visual impairment obscures the distinction.

Once separated, the account becomes coherent. The visual pathways may indeed have been spared while photophobia was present, provided blood and necrotic material reached the subarachnoid space. Meningeal involvement would then account, through a single mechanism, for the headache, the vomiting, the intolerance of light and the high fevers recorded, the last of which need not have been infectious. This is worth stating, since it was fever that led Maestro Moyse towards consumption.

A second point concerns the terminal mechanism, which the article does not specify. Apoplexy is named as the cause of death, but haemorrhagic infarction of the pituitary does not itself kill a woman of twenty‐three. Acute corticotroph failure does. Sudden loss of ACTH secretion produces hypotension, hyponatraemia and hypoglycaemia, with progressive encephalopathy and circulatory collapse, which is why immediate glucocorticoid replacement remains the priority in severe apoplexy today [3]. This also offers the most parsimonious reading of the confusion and of the episodes described as hallucination: a delirium of metabolic origin rather than a focal perceptual disturbance. The single surviving letter permits no stronger claim than that.

Finally, the collapse at the ball is treated as a precipitant, the mechanical stress of dancing hastening haemorrhage. The reverse ordering is at least as economical. The collapse and epistaxis may have been the first observable manifestation of an apoplexy already under way, the ball being merely where it declared itself. This requires no additional precipitating event and leaves the central hypothesis intact.

None of this is verifiable, and the reconstruction rests on a single archival letter and on a portrait series at least two items of which postdate her death. But if the diagnosis is granted, the sequence it implies is more specific than a list of compatible symptoms: sellar floor erosion with sphenoidal extension for the epistaxis and rhinorrhoea; subarachnoid spread for the meningeal syndrome; and acute pituitary failure for the death itself.

## Author Contributions

**Alexis Demas:** conceptualization, investigation, writing – original draft, writing – review and editing, visualization, validation, methodology, software, formal analysis, project administration, resources, supervision, data curation.

## Funding

The author has nothing to report.

## Ethics Statement

The author has nothing to report.

## Conflicts of Interest

The author declares no conflicts of interest.

## Data Availability

The data that support the findings of this study are available from the corresponding author upon reasonable request.
